# A novel approach to minimize false discovery rate in genome-wide data analysis

**DOI:** 10.1186/1752-0509-7-S4-S1

**Published:** 2013-10-23

**Authors:** Yuanzhe Bei, Pengyu Hong

**Affiliations:** 1 Department of Computer Science, Brandeis University, 415 South St., MA 02453, USA

## Abstract

**Background:**

High-throughput technologies, such as DNA microarray, have significantly advanced biological and biomedical research by enabling researchers to carry out genome-wide screens. One critical task in analyzing genome-wide datasets is to control the false discovery rate (FDR) so that the proportion of false positive features among those called significant is restrained. Recently a number of FDR control methods have been proposed and widely practiced, such as the Benjamini-Hochberg approach, the Storey approach and Significant Analysis of Microarrays (SAM).

**Methods:**

This paper presents a straight-forward yet powerful FDR control method termed miFDR, which aims to minimize FDR when calling a fixed number of significant features. We theoretically proved that the strategy used by miFDR is able to find the optimal number of significant features when the desired FDR is fixed.

**Results:**

We compared miFDR with the BH approach, the Storey approach and SAM on both simulated datasets and public DNA microarray datasets. The results demonstrated that miFDR outperforms others by identifying more significant features under the same FDR cut-offs. Literature search showed that many genes called only by miFDR are indeed relevant to the underlying biology of interest.

**Conclusions:**

FDR has been widely applied to analyzing high-throughput datasets allowed for rapid discoveries. Under the same FDR threshold, miFDR is capable to identify more significant features than its competitors at a compatible level of complexity. Therefore, it can potentially generate great impacts on biological and biomedical research.

**Availability:**

If interested, please contact the authors for getting miFDR.

## Background

FDR control is a statistical approach to correct multiple comparisons in dealing with multiple hypothesis testing problems. It has now been widely practiced in analyzing genome-wide datasets generated by high-throughput technologies, such as DNA microarray and RNA-Seq, which allows users to simultaneously screen the activities of tens of thousands of genes. These high-throughput datasets require careful analysis to identify a subset of interesting molecular features for follow-up experiments. It is always desired to maximizing findings in data. In the meantime, it should be realized that follow-up experiments can be costly in both time and money. Therefore it is important to control the proportion of wrongly called features among those selected (i.e., FDR).

FDR was first introduced by Benjamini and Hochberg [1] and was later improved by the Storey procedure [2,3]. As two of the mainstream FDR controlling methods, the BH procedure fixes the error rate and then estimates its corresponding rejection region while the Storey procedure fixes the rejection region and then estimates its corresponding error rate. Efron and his colleagues framed the FDR control problem as a Bayesian problem, and showed that both the BH and Storey approaches are special cases [4-6]. Assuming that the same rejection region is used for each independent test, and the test statistics come from a random mixture of null and alternative distributions, the BH approach, the Storey approach and the Efron's Bayesian approach can be connected with a mixture model of null statistics and alternative statistics weighted by a factor representing the prior probability of getting true nulls. The BH approach simply assumes that the prior probability of true null is equal to 1, which makes it the most conservative one among the three. The Storey approach considers estimating the prior probability of true null. The Efron approach uses empirical Bayesian analysis to further estimate posterior probability of true null based on the prior probability. The BH, Storey and Efron approaches all estimate FDR by taking the *p*-values of individual features calculated by some sorts of hypothesis tests. The *t*-test [7] and the Wilcoxon ranksum test [8] (also known as the Mann-Whitney U test, referred as ranksum test in the rest of the paper for conciseness) are two of the most well-known tests for calculating the *p*-values of individual features.

Significance Analysis of Microarrays (SAM) [9] is another widely applied technique for calling features that behave significantly differently between two conditions. Different from the BH, Storey and Efron approaches, SAM uses a nonparametric method to estimate FDR instead of relying on *p*-values directly. SAM generates a large number of permutation controls, and the expected number of false positives can be estimated by counting the number of permuted statistics beyond a certain cut-off. Although SAM performed better than the BH and Storey approaches on many datasets in our practices, we found that SAM's results were not optimal in many cases. This is mainly because SAM decides the cut-offs based on the differences between the observed statistics (original statistics) and the expected statistics (averaged statistics from permuted measurements) instead of the estimated FDR, which does not guarantee the lowest FDR.

To address this problem, we developed miFDR - an advanced significance analysis method for optimizing FDR when the number of desired significant features is fixed. A preliminary version of miFDR was published in [10]. In this paper, we provide theoretical explanations and supports for miFDR, and generate more experimental results to demonstrate that miFDR empirically outperforms SAM, the BH approach and the Storey approach. In particular, the simulation test results showed that miFDR was capable of identifying more significant features with its true FDRs consistently bounded by the estimated FDRs. In addition, the true and estimated FDRs of miFDR were lower than those of the other three methods. Furthermore, when applied to real DNA microarray datasets, miFDR was able to identify more biologically relevant genes than other methods.

## Methods

### FDR under the Bayesian framework

FDR is defined as the expected proportion of incorrectly rejected null hypotheses among all rejected null hypotheses. It can be represented as a conditional probability *P*(*H *= 0|*d *∈ Γ ), where Γ denotes the rejection region for the statistic variable *d*. Applying the Bayes theorem, the above definition can be written as

(1) $$\text{FDR}\left(d,\text{Γ}\right)=P\;\left(H=0|d\in \text{Γ}\right)=\frac{P\;\left(H=0\right)P\;\left(d\in \text{Γ}|H=0\right)}{P\;\left(d\in \text{Γ}\right)}$$

where *P *(*d *∈ Γ) = *P *(*H *= 0) *P *(*d *∈ Γ|*H *= 0) + *P *(*H *= 1) *P *(*d *∈ Γ|*H *= 1).

The estimation of *P *(*d *∈ Γ|*H *= 0) is not straightforward. However, if the null distribution of *d *is known, the above term can be calculated by *p*-value: *P *(*p *≤ *γ *| *H *= 0) where *p *is the *p*-value of *d *and *γ *is the corresponding *p*-value cut-off to reject *H *= 0. The BH and Storey approaches are based on this idea. Assuming that the null distribution of *p*-values is uniform between 0 and 1, we have

(2) $$\text{FDR}\left(p,\gamma \right)=\frac{P\left(H=0\right)P\left(p\leq \gamma |H=0\right)}{P\left(p\leq \gamma \right)}=\frac{P\left(H=0\right)\gamma }{P\left(p\leq \gamma \right)}$$

The term *P *(*p *≤ *γ*) can be estimated in an empirical way as the proportion of features whose *p*-values are bounded by the *p*-value cut-off *γ*, namely $\hat{P}\left(p\leq \gamma \right)=\#\left\{p_{i}\leq \gamma \right\}/\#\left\{p_{i}\right\}=\#\left\{p_{i}\leq \gamma \right\}/M$, where #(*p_i _*≤ *γ*) denotes the number of *p*-values bounded by the cut-off *γ*, and #{*p_i_*} denotes the total amount of *p*-values which is equivalent to the total number of features *M*. The BH approach simply assumes that *P*(*H *= 0) = 1 while the Storey approach estimates *P*(*H *= 0) empirically [2]. SAM adopts the same method to estimate *P*(*H *= 0) as the Storey approach [11].

### SAM

Different from the BH and Storey approaches, SAM does not assume the distributions of the test statistics. In addition, it introduces corrections to *t*-statistic and ranksum statistic so that the distribution of the corrected statistics is independent from the levels of feature values. Both *t*-statistic and ranksum statistic can be represented as a difference score *r_i _*divided by the corresponding standard deviation *s_i _*: *r_i_*/*s_i_*. In particular, let *X *and *Y *be two groups of samples with *N_X _*and *N_Y _*samples, respectively. The traditional *t*-statistic has $r_{i}={\bar{X}}_{i}-{\bar{Y}}_{i}$ and $s_{i}=\{\left[\underset{x_{im}\in X}{\sum }{\left(x_{im}-{\bar{X}}_{i}\right)}^{2}+\underset{y_{in}\in Y}{\sum }{\left(y_{in}-{\bar{Y}}_{i}\right)}^{2}\right]\;\left(1/N_{X}+1/N_{Y}\right)/(N_{X}+N_{Y}-2\}{}^{1/2}$; and the traditional ranksum statistic has $r_{i}={\bar{R}}_{i}^{X}-N_{X}\left(N_{X}+N_{Y}+1\right)/2$ and $s_{i}=N_{X}N_{Y}\left(N_{X}+N_{Y}+1\right)/12$, where ${\bar{R}}_{i}^{X}$ is the sum of the ranks of the *i*-th feature from *X *(the measurements from *X *and *Y *are merged and then ranked from lowest to highest). They share one major drawback: the estimation of the standard deviation *s_i _*is very unstable when the sample size is relatively small, which is very common in studies involving high-throughput technologies. In addition, the distributions of the test statistics vary with respect to the levels of feature values, which makes it difficult to compare features with different value levels. To address these problems, SAM adds a factor *s*_0 _to the denominator *s_i _*to reduce the variance of the corrected statistic *d_i _*= *r*_i_/(*s_i _*+ *s*_0_) (referred as *d*-value in the rest of paper for conciseness). In practice, SAM chooses the value of *s*_0 _from the pool of all {*s_i_*} so that the variance of *d_i _*is minimized. The goal is to make the variance of *d_i _*independent to the expression level [11]. We found the corrected statistics useful in analyzing many datasets in our research.

Since the null distributions of the corrected statistics are unknown, SAM uses permutations of the replicates to estimate FDR. Given a particular rejection region Γ, SAM generates *B *permutations of the original measurements and estimates *P *(*d *∈ Γ|*H *= 0) as the median of ${\left\{P_{b}\left(\hat{d}\in \text{Γ}\right)\right\}}_{b=1\ldots B}$, where $\hat{d}$ denotes the *d*-values in the *b*-th permutation. SAM estimates *P *(*H *= 0) in the same way as the Storey approach does [11].

In SAM, the rejection region Γ is determined by a positive cut-off *τ*^+ ^> 0 and a negative cut-off *τ*^- ^< 0. The corresponding rejection regions are ${\text{Γ}}^{+}=\left\{d|d>\tau^{+}>\;0\right\}$ and ${\text{Γ}}^{-}=\left\{d|d<\tau^{-}<0\right\}$, respectively. A feature is called "significant positive" if its *d*-value is greater than *τ*^+^, or "significant negative" if its *d*-value is smaller than *τ*^-^. The total rejection region is

(3) $$\text{Γ}\left(\tau^{+},\tau^{-}\right)={\text{Γ}}^{+}\cup {\text{Γ}}^{-}=\left\{d|d>\tau^{+}>0\;or\;d<\tau^{-}<0\right\}$$

To decide *τ*^+ ^and *τ*^-^, SAM introduces a Δ-index which is calculated as follows. First, the features are sorted in ascending order based on their original *d*-values. Let $\left\{d_{i}^{*}\right\}$ denote the *d*-values of the sorted features. Then the *d*-values obtained from the permutated replicates are sorted and used to estimate $E\;\left[{\hat{d}}_{i}^{*}\right]$. Finally, the Δ-value of the *i*-th feature is calculated as ${\text{Δ}}_{i}=d_{i}^{*}-E\left[{\hat{d}}_{i}^{*}\right]$. Given a user-defined threshold Δ_0_, SAM searches in the ascending order of Δ-values and decides $\tau^{+}=d_{k}^{*}$, where *k *is the index of the first feature satisfying Δ*_k _*≥ Δ_0_. Similarly, SAM searches in the descending order of Δ-value and decides the negative cut-off as $\tau^{-}=d_{l}^{*},$ where *l *is the index of the first feature satisfying Δ*_l _*≤ -Δ_0_.

**Figure 1:**
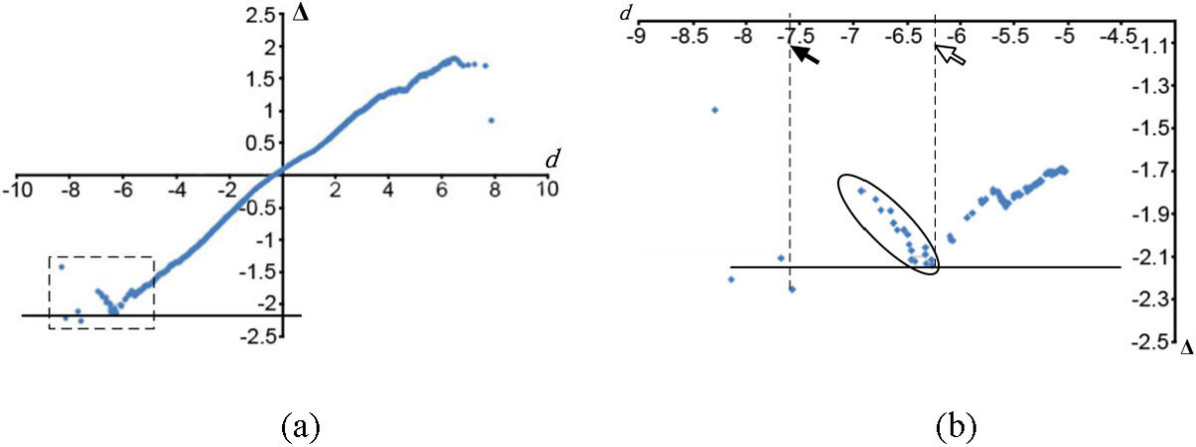
**The problem of relying on **Δ**-values to call features**. An example showing the problem of using Δ-values to decide the *d*-value cut-offs. The *x*-axis indicates *d*-values and the *y*-axis indicates Δ-values. (b) is the blow-out of the dashed rectangle region in (a). Two *d*-value cut-offs are indicated by the solid arrow and the open arrow respectively in (b) (see main text for a detailed explanation). The solid arrow marks the negative *d*-value cut-off *τ^− ^*corresponding to the Δ-value indicated by the black line.

It is obvious that the behaviour of Δ-values has a great impact on SAM's results. We observed that Δ-values were not always monotonic with respect to *d*-values, which can greatly limit SAM's performance. To illustrate this, we used a Gene Expression Omnibus (http://www.ncbi.nlm.nih.gov/geo/) dataset GDS3661 [12] as an example. Shown in Figure 1(a), the monotony does not hold at both ends of the curve, especially the lower end. In Figure 1(b), a group of features, whose *d*-values ranged from -6.25 to -7, are circled by an ellipse. Let Δ^* ^denote the smallest Δ-values of these circled features. Given any small constant *δ *> 0, if we change the threshold from Δ* - *δ *to Δ* + *δ*, the negative cut-off *τ*− will jump significantly from -6.25 (marked by the white arrow) to -7.6 (marked by the black arrow). This means either all of those circled features will be called as significant simultaneously, or none of them will be called. No valid threshold allows a subset of them to be called significant even though FDR can be improved by doing so. Therefore it is not always reliable to determine *d*-value cut-offs based on Δ-values. This inspired us to develop miFDR which relies only on *d*-values and will be explained below.

### Minimize FDR - miFDR

If we would like to select *N *> 0 significant features (*N*^+ ^positive significant features and *N*^- ^negative significant features), there are *N *+ 1 different possible options for choosing (*N*^+^, *N*^-^), i.e. (0, *N*), (1, *N *- 1), (2, *N *- 2), ···, (*N*, 0). These options assume: If two *d*-values are of the same sign, the one with larger absolute value is more significant. SAM does not explore all of these options, and hence its results can be sub-optimal. We designed a straight-forward algorithm (pseudo codes in **Algorithm 1**) called miFDR to explore all *N *+ 1 options and report the one with the lowest estimated FDR. Mathematically, this idea can be expressed as

(4) $$\left(N_{+}^{*},N_{-}^{*}\right)=\underset{N^{+}+N^{-}=N}{\text{arg }\text{min}}\;\text{FDR}\left(N^{+},N^{-}\right)$$

where FDR(*N*^+^, *N*^-^) indicates the FDR of choosing *N*^+ ^positive significant features and *N*^- ^negative significant features. Given a FDR cut-off Ψ ∈ (0,1), we can find the global optimal N*

(5) $$\begin{array}{c} N^{*}=\text{max}\left(N|\text{FDR}\left(N_{+}^{*},N_{-}^{*}\right)<\text{Ψ}\right)\\ \text{where}\;N_{+}^{*}\;\text{and}\;N_{-}^{*}\;\text{are}\;\text{obtained}\;\text{by}\;\text{solving }\text{eq}.\;(4) \end{array}$$

**Theorem 1**: Given a FDR cut-off Ψ ∈ (0,1), miFDR always finds the maximum number of significant features.

**Proof**: Eq. (5) is equivalent to

(6) $$N^{*}=\;\text{min}\left(N|\text{FDR}\left(N_{+}^{*},N_{-}^{*}\right)\geq \text{Ψ}\right)-1$$

According to eq. (4), for ∀*N*^+^, *N*^− ^≥ 0 *s.t. N*^+ ^+ *N*^− ^= *N*, $\text{FDR}\left(N_{+}^{*},N_{-}^{*}\right)\leq \text{FDR}\left(N^{+},N^{-}\right)$. This means: If $\text{FDR}\left(N_{+}^{*},N_{-}^{*}\right)\geq \text{Ψ}$, then ∀*N*^+^, *N*^− ^≥ 0 *s.t. N*^+ ^+ *N*^− ^= *N*, we have $\text{FDR}\left(N^{+},N^{-}\right)\geq \;\text{FDR}\left(N_{+}^{*},N_{-}^{*}\right)\geq \text{Ψ}$. Therefore, eq. (6) can be written as

(7) $$N^{*}=\text{min}\left(N|\forall N^{+},N^{-}\geq 0\;s.t.\;N^{+}+N^{-}=N\&\text{FDR}\left(N^{+},N^{-}\right)\geq \text{Ψ}\right)-1$$

This is equal to

(8) $$N^{*}=\text{max}\left(N|\exists N^{+},N^{-}\geq 0\;s.t.\;N^{+}+N^{-}=N\&\text{FDR}\left(N^{+},N^{-}\right)<\text{Ψ}\right)$$

Eq. (8) indicates that miFDR always finds the maximum number of features given a specific FDR cut-off.

The maximum number of features that SAM is able to call under the FDR cut-off Ψ can be written as:

(9) $$N_{SAM}^{*}=\text{max}\left(N|\exists {\text{Δ}}_{0}\;s.t.\;N_{{\text{Δ}}_{0}}^{+}+N_{{\text{Δ}}_{0}}^{-}=N\&\text{FDR}\left(N_{{\text{Δ}}_{0}}^{+},N_{{\text{Δ}}_{0}}^{-}\right)<\text{Ψ}\right)$$

where $N_{{\text{Δ}}_{0}}^{+}$ and $N_{{\text{Δ}}_{0}}^{-}$ respectively are the numbers of positive and negative features called significant by the positive and negative *d*-value cut-offs decided by Δ_0_. It should be noted that users need to manually try several Δ-value cut-offs to find the best Δ_0_. It is obvious that $\left(N_{{\text{Δ}}_{0}}^{+},N_{{\text{Δ}}_{0}}^{-}\right)$ is a special case of $\left(N^{+},N^{-}\right)$ in eq. (8). Hence SAM only explores a subset of options considered by miFDR mainly because SAM does not directly tune *d*-value cut-offs. Instead, SAM control *d*-value cut-offs via Δ_0_. Thus the best result of SAM is bounded by the best result of miFDR.

Algorithm 1: [*fdr, feature^+^, feature^-^*] = ***miFDR ***$\left(\left\{d_{i}\right\},\;\left\{{\hat{d}}_{i,b}\right\},N\right)$

***Input: ***{*d_i_*} - the original *d*-values; $\left\{{\hat{d}}_{i,b}\right\}$ - the *d*-values in permutations; *N *- the number of desired significant features.

1) Initialize *fdr ← *∞, *N*^+ ^*← *0, and *N*^- ^*← *0.

2) Sort {*d_i_*} in the ascending order to obtain $\left\{d_{i}^{*}\right\}.$

3) For (*n *= 0; *n *≤ *N*; *n*++)

a) Select *n *positive significant features and *N - n *negative significant features.

b) Define the corresponding rejection region

$$\text{Γ}\left(d_{M-n}^{*},\;d_{N-n+1}^{*}\right)=\left\{d|d>d_{M-n}^{*}\;or\;d<d_{N-n+1}^{*}\right\}$$

c) Estimate FDR "*cFDR*" for above rejection region using $\left\{{\hat{d}}_{i,b}\right\}$.

d) If *cFDR *<*fdr*, then *fdr *= *cFDR*, and update *N*^+ ^← *n *and *N*^- ^← *N *- *n*.

4) Let *feature*^+ ^= the indexes of *N*^+ ^features with the largest *d*-values; and *feature^- ^*as the indexes of *N*^- ^features with the smallest *d*-values.

***Output: ****fdr *- the estimated FDR; *feature*^+ ^- the indexes of positive significant features; *feature^- ^*- the indexes of negative significant features.

### Computational complexity of miFDR

Assume that a dataset is composed of *W *samples, each sample has *M *features, and the samples are permutated *P *times to generate the control. It takes O(*WMP*) for miFDR to compute the permuted statistics (the computation time for one feature in one permutation is proportional to the sample size *W*), and the computation time for the original statistics can be ignored because P≫1. Once the original and permuted statistics are computed, miFDR can be applied to achieve two typical goals:

• **Minimize FDR when finding *N *significant features**: In this case, miFDR needs to explore *N *+ 1 options. For each option, namely a given (*N*^+^, *N*^-^) pair, the expected computation time for miFDR to estimate FDR is O(*MP*) because it has to go through the entire permutation matrix. Thus, the computational complexity for miFDR applied to this goal is O(*NMP*).

• **Find the maximal number of significant features given a FDR cut-off**: In this case, miFDR needs to examine up to a certain number *M_ρ _*(0 <*M_ρ _*≤ *M*) in order to find the best result yielding the required FDR cut-off. In the worst-case scenario, miFDR has to estimate FDRs for all $\frac{1}{2}{M_{\rho }}^{2}$ possible (*N*^+^, *N*^-^) airs that satisfy *N*^+ ^+ *N*^− ^= 1,2, ..., *M_ρ_*. If not well implemented, the worst computational complexity for doing this is $O\left({M_{\rho }}^{2}MP\right)$, which is much worse than that of the first goal. However, we noticed that both *N*^+ ^and *N*^- ^can only be chosen from 0, 1, 2, ...*M_ρ_*. Thus, miFDR can be implemented in a very efficient way as below. We can use the permutated measurements to calculate in advance the one-sided false positives for *N*^+ ^= 0,1,2, ..., *M_ρ _*and *N*^− ^= 0,1,2, ..., *M_ρ _*in each permutation. This will take O(*M_ρ_MP*) in total. Then, to evaluate a given (*N*^+^, *N*^-^) pair, all we need to do is simply combine the pre-computed false positives for *N*^+ ^and those for *N*^- ^in each permutation, and then calculate the median of the combined false positives in O(*P*). Hence, it will take miFDR $O\left({M_{\rho }}^{2}P\right)$to cover all $\frac{1}{2}{M_{\rho }}^{2}$options. Since *M_ρ _*≤ *M*, miFDR has a computational complexity of O(*M_ρ_MP*) to check up to *M_ρ _*features, which is comparable to that of the first goal if the desired number of features *N *~ *M_ρ_*. Based on the above idea, we designed **Algorithm 2**. In practice, the value of *M_ρ _*can be easily tuned by users. By default, we set it to 1000, which worked well in practice so far, and the calculation can be finished in a few minutes. Nevertheless, we theoretically proved that *M_ρ _*can be determined automatically and efficiently (see Theorem S1 in Additional File 1 Appendix C).

Algorithm 2: [*N, feature^+^, feature^-^*] = ***miFDR2 ***$\left(\left\{d_{i}\right\},\;\left\{{\hat{d}}_{i,b}\right\},\text{Ψ},M_{\rho }\right)$

***Input: ***{*d_i_*} - the original *d*-values; $\left\{{\hat{d}}_{i,b}\right\}$- the *d*-values in permutations; Ψ - target FDR cut-off; *M_ρ _*- the expected number of significant features to be tested.

1) Initialize *fdr ← *∞, *N*^+ ^← 0, and *N*^- ^← 0.

2) Calculate *P*(*H *= 0).

3) Sort {*d_i_*} in the ascending order to obtain $\left\{d_{i}^{*}\right\}.$

4) For (*k *= 0; *k *≤ *M_ρ_*; *k*++)

a) $TP_{k}^{+}=$ the number of the original *d*-values larger than $d_{M-k}^{*}.$

b) $TP_{k}^{-}=$ the number of the original *d*-values smaller than $d_{k+1}^{*}.$

c) For (*b *= 0; *k *≤ *P*; *b*++)

i. $FP_{k,b}^{+}=$ the number of the *d*-values in *b*-th permutation that are larger than $d_{M-k}^{*}.$

ii. $FP_{k,b}^{-}$ = the number of the *d*-values in *b*-th permutation that are smaller than $d_{k+1}^{*}.$

5) For (*k *=*M_ρ_*; *k *≥ *0*; *k *−−)

a) Initialize $\hat{cFDR}\leftarrow \infty ,{\hat{N}}^{+}\leftarrow 0,\;and\;{\hat{N}}^{-}\leftarrow 0$

b) For (*n *= 0; *n *≤ *k*; *n*++)

i. The estimated FDR can be obtained efficiently as follows:

$$cFDR=\frac{\text{median}\left({\left\{FP_{n,b}^{+}+FP_{k-n,b}^{-}\right\}}_{b=1\ldots B}\right)\cdot P\left(H=0\right)}{TP_{n}^{+}+TP_{k-n}^{-}}$$

ii. *If *$cFDR<\hat{cFDR}$, then $\hat{cFDR}\leftarrow \;cFDR$, ${\hat{N}}^{+}\leftarrow n$ and ${\hat{N}}^{-}\leftarrow k-n$.

c) $If\;\hat{cFDR}<\text{Ψ}$, then $fdr\leftarrow \hat{cFDR}$, $N^{+}\;\leftarrow {\hat{N}}^{+},N^{-}\leftarrow {\hat{N}}^{-}$, and *N *← *k*. Jump to (6).

6) Let *feature*^+ ^= the indexes of *N*^+ ^features with the largest *d*-values; and *feature*^- ^= the indexes of *N*^- ^features with the smallest *d*-values.

***Output: ****N - *the maximum number of significant features satisfying the FDR cut-off Ψ; *feature^+ ^*- the indexes of positive significant features; *feature^- ^*- the indexes of negative significant features.

## Results

We compared the miFDR approach to SAM (v4.0), the BH approach and the Storey approach on both simulation test and real microarray data analysis. We selected two-sided *t*-test *p*-values for the BH and Storey approaches (implemented in MATLAB Bioinformatics Toolbox v4.2) because we found two-sided *t*-test has better performance than one-sided *t*-test, one-sided ranksum test and two-sided ranksum test. The results showed that miFDR outperformed the other three methods in a wide range of FDR cut-offs.

### Simulation test

We ran the simulation test 1000 times, which were designed to have enough complexity to extensively test different FDR controlling methods. In each run, a dataset was simulated according to the distributions in Table 1. Each simulation dataset contains 16 samples in total, 8 samples in each group. Each sample has 10400 features: 10000 null hypothesis features + 400 alternative hypothesis features. Out of 10000 null hypothesis features, 5000 features follow standard normal distribution and the rest follow uniform distribution in range $\left[-\sqrt{3,}+\sqrt{3}\right].$ And 400 alternative hypothesis features follow a mixture of multiple distributions described in Table 1.

**Table 1:** Null and alternative hypotheses in simulated datasets

Category	# of features	Group 1	Group 2
1	5000	Gaussian with mean = 0 and variance = 1	Gaussian with mean = 0 and variance = 1
2	5000	Uniform in range $\left[-\sqrt{3,}\sqrt{3}\right]$	Uniform in range $\left[-\sqrt{3,}\sqrt{3}\right]$
3	50	Gaussian with mean = 0 and variance = 1	Gaussian with mean = -2 and variance = 1
4	150	Gaussian with mean = 0 and variance = 1	Gaussian with mean = 1 and variance = 1
5	150	Uniform in range $\left[-\sqrt{3,}\sqrt{3}\right]$	Uniform in range $\left[1-\sqrt{3,}1+\sqrt{3}\right]$
6	50	Uniform in range $\left[-\sqrt{3,}\sqrt{3}\right]$	Uniform in range $\left[1.5-\sqrt{3,}1.5+\sqrt{3}\right]$

Each simulation dataset has 10400 features, which are divided into six groups. The number of features in each group and the corresponding distributions are listed below. Category 1 and 2 are for null hypotheses features and category 3, 4, 5 and 6 are for alternative features. Particularly in Category 2, we use uniform distribution in range $\left[-\sqrt{3,}\sqrt{3}\right]$ to make the variance equal to 1.

In each simulation, every approach produced a curve describing the estimated FDR *vs*. the number of significant features. Those 1000 curves were then averaged with respect to the number of significant features. Since the ground-truth was known, we were able to calculate the true FDR and derive the averaged curve to show true FDR *vs*. the number of significant features for each approach.

**Figure 2:**
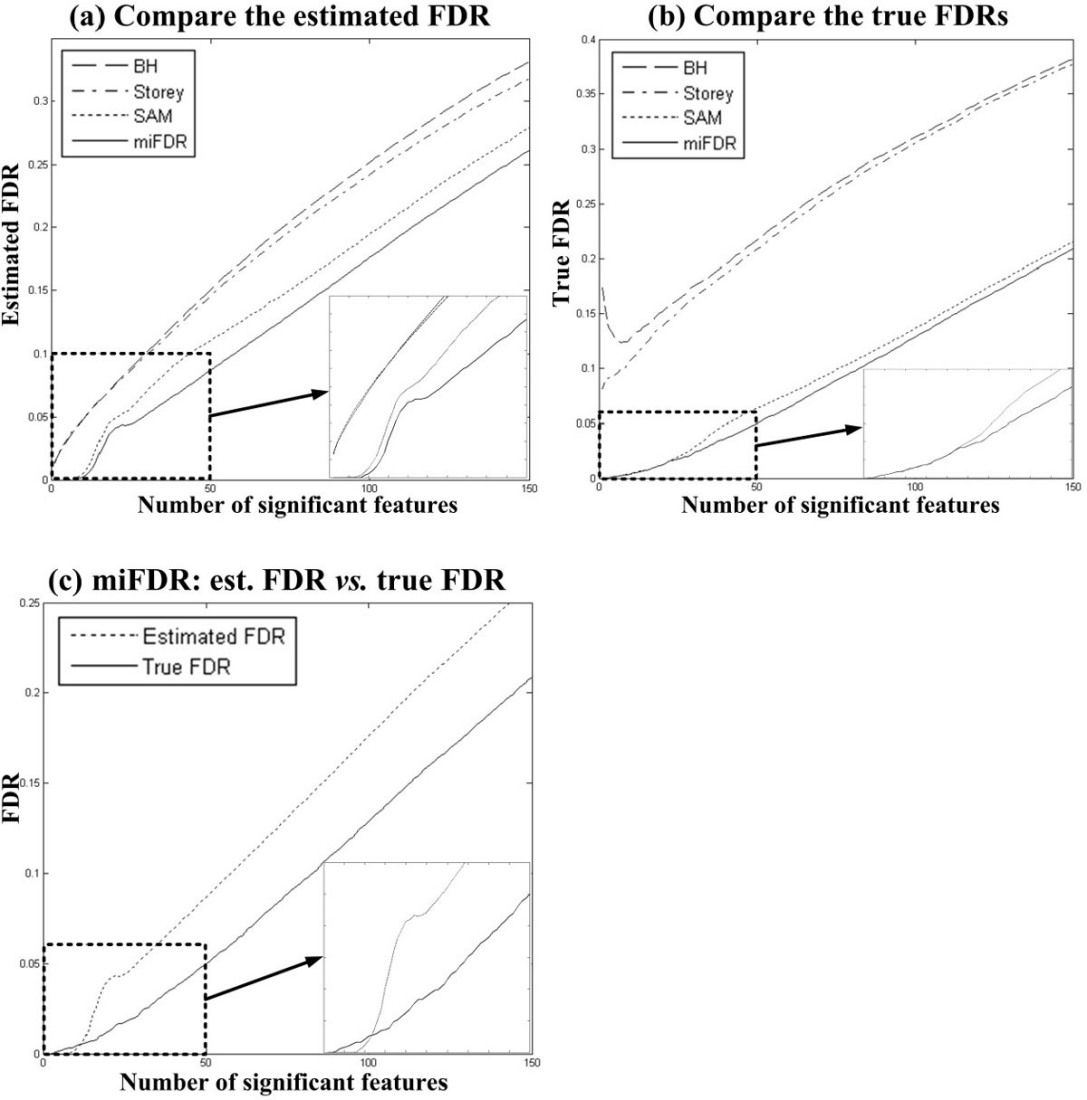
**Simulation result I**. Compare the average performance of BH, Storey, SAM and miFDR on 1000 simulation runs. In each plot, a blow-out of the curve segment in the dashed rectangle is shown at the bottom-right corner for clearer illustration. **(a) **Compare the estimated FDRs among four approaches. The performance of miFDR is the best. **(b) **Compare the true FDRs. The performance of miFDR is the best. **(c) **Compare the estimated FDRs and the true FDRs of miFDR. The true FDR curve of miFDR is well bounded by its estimated FDR curve, which indicates that miFDR does not under-estimate the number of falsely called significant features.

As expected, miFDR consistently called more significant features than SAM at the same estimated FDR levels (see Figure 2a). In particular, at FDR cut-off level 0.05, miFDR identified 19.64 features on average, 17.61% more than the average 16.18 features identified by SAM. Paired *t*-test showed that the results of miFDR was significantly better than those of SAM (*p-*value = 6.04e-73). In addition, the true FDR curve of miFDR was consistently bounded by that of SAM (see Figure 2b). This means miFDR made less false calls than SAM did. Finally, the true FDR curve of miFDR was well bounded by its estimated FDR curve (see Figure 2c).

**Figure 3:**
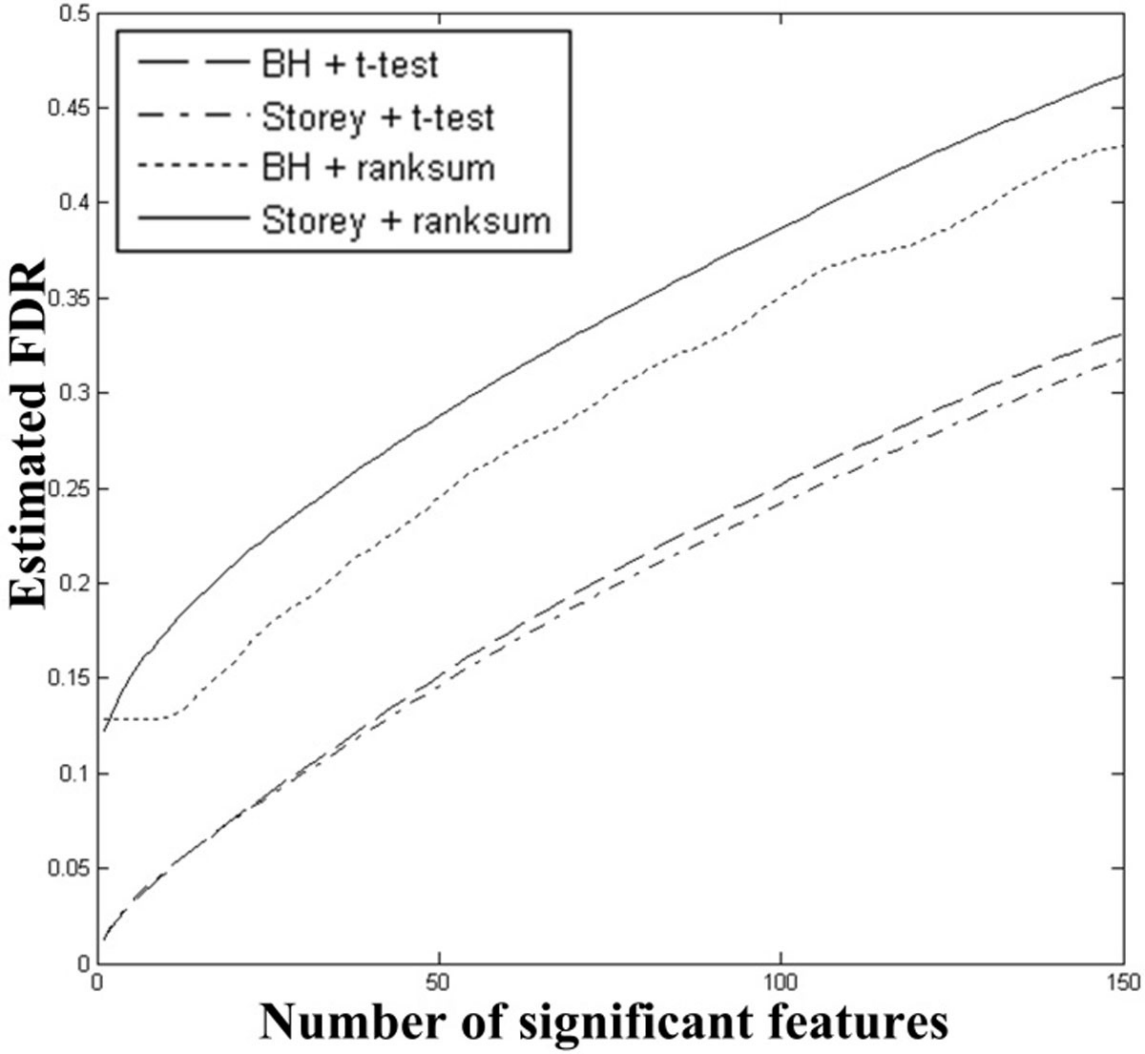
**Simulation result II**. Compare the average performance of four approaches: 1) BH using *t*-test, 2) Storey using *t*-test, 3) BH using ranksum and 4) Storey using ranksum on 1000 simulation runs. The performance of BH using *t*-test and Storey using *t*-test are much better than those of BH using ranksum and Storey using ranksum.

The BH and Storey approaches were also included in the comparison. But their performance was much worse than miFDR and SAM (Figure 2a &2b), with two reasons: Firstly, they consistently identified fewer significant features than miFDR and SAM did at the same FDR levels. Secondly, their true FDRs are much higher than those of miFDR and SAM when calling the same numbers of significant features. Such worse performance can be because 50% null features follow uniform distributions. However the BH and Storey approaches used *t*-test *p*-values, which assume Gaussian distributions. When ranksum *p*-values were used in the BH and Storey approaches, the results were even worse (see Figure 3).

We also ran the simulation test with sample size 6 *vs*. 6 and 10 *vs*. 10. The results resonated the above findings (see the Additional File 1 Appendix A & B).

### Analyze DNA microarray datasets

To further demonstrate that miFDR has high performance in practice, we compared miFDR, SAM, the BH and Storey approaches on a couple of public DNA microarray gene expression datasets obtained from Gene Expression Omnibus (GEO, http://www.ncbi.nlm.nih.gov/geo/). The results (see Table 2) clearly showed that miFDR significantly outperformed the other three approaches.

**Table 2:** Numbers of detected significant genes under FDR cut-off = 0.05

Dataset	miFDR	SAM	BH - *t*-test	Storey - *t*-test	BH - ranksum	Storey - ranksum
GDS1517*	610	546	20	29	0	0
GDS2154	684	292	150	300	0	0
GDS2414	322	286	102	115	0	0
GDS2470	104	58	4	4	0	0
GDS2552	787	741	245	508	0	0
GDS2765	177	112	10	20	0	0
GDS2778	734	701	57	108	0	13
GDS3087	74	15	2	4	0	0
GDS3132	453	420	163	205	0	0
GDS3295**	342	267	149	319	0	0
GDS3395	463	425	98	98	210	56
GDS3407***	283	241	1	1	0	0
GDS3518	450	420	23	43	0	0
GDS3663	419	235	0	0	0	0

Numbers of detected significant genes under FDR cut-off = 0.05. Three out of total 14 datasets have more than two classes. In each of those datasets, we select two classes as the following. *GDS1517: wild type/control (5 samples) *versus *wild type/low fat, high carbohydrate (5 samples). **GDS3295: GV oocyte/66 weeks (4 samples) *versus *GV oocyte/6 weeks (4 samples). ***GDS3407: wild type/control (4 samples) *versus *wild type/PFOA (4 samples).

Two of those datasets happen to be related to hypertension: GDS3661 and GDS3689, which caught our attentions. Hypertension accounts for about 25% of heart failures [13]. If uncontrolled, hypertension can cause various changes in myocardial structure, conduction system and coronary vasculature of the heart, which can further cause the development of left ventricular hypertrophy, atherosclerosis and other complications. It has been proved by both experimental animal and clinical studies that left ventricular hypertrophy could induce myocardial ischemia [3], and eventually result in large-scale programmed cell death and heart failure. We therefore want to see if genes called by miFDR in these datasets are indeed biologically relevant. GDS3661 was generated to investigate the molecular activity underlying the onset hypertensive heart failure, by profiling left ventricular samples from rats with spontaneously hypertension [14]. It used Affymetrix Rat Genome 230 2.0 Array to compare the gene expression levels of 6 heart failure rats (HF-rats) with those of 6 rats without heart failure (Control-rats). Hypertension has been proved by many studies to be highly related to environmental pollution, especially diesel exhaust exposure [15-19]. To discover molecular links between hypertension and diesel exhaust exposure, GDS3689 was generated by profiling samples from rats exposed to diesel exhaust particles [20] using Affymetrix Gene Chip Rat 230A microarray. It compared 4 rats exposed to diesel exhaust particles (DE-rats) with 4 rats without exposure (Control-rats). GDS3689 contains samples of both hypertensive rats and healthy rats. In this paper, we only analyzed the samples of healthy rats.

#### GDS3661

We set the FDR cut-off as 0.05, and compared the number of probe sets called significant by the BH approach, the Storey approach, SAM, and miFDR. Using either *t*-test or ranksum test, both the BH approach and the Storey approach failed to identify any significant probe set. At the same FDR cut-off, miFDR identified 210 probe sets versus 129 probe sets identified by SAM.

The probe set lists detected by SAM and miFDR were submitted to DAVID [21,22] for gene ontology (GO) enrichment analysis. The result showed that miFDR was better than SAM in identifying genes with functions closely related to phenotypic changes from compensated hypertrophy to systolic heart failure. Some typical GO categories are: GO-0007179: transforming growth factor beta receptor signaling pathway (miFDR matched 4 genes *vs*. SAM matched 3 genes), GO-0010647: positive regulation of cell communication (miFDR 10 genes *vs*. SAM 5 genes), GO-0012501: programmed cell death (miFDR 9 genes *vs*. SAM 7 genes), GO-0033554: cellular response to stress (miFDR 10 genes *vs*. SAM 5 genes), and GO-0040008: regulation of growth (miFDR 9 genes *vs*. SAM 3 genes).

**Figure 4:**
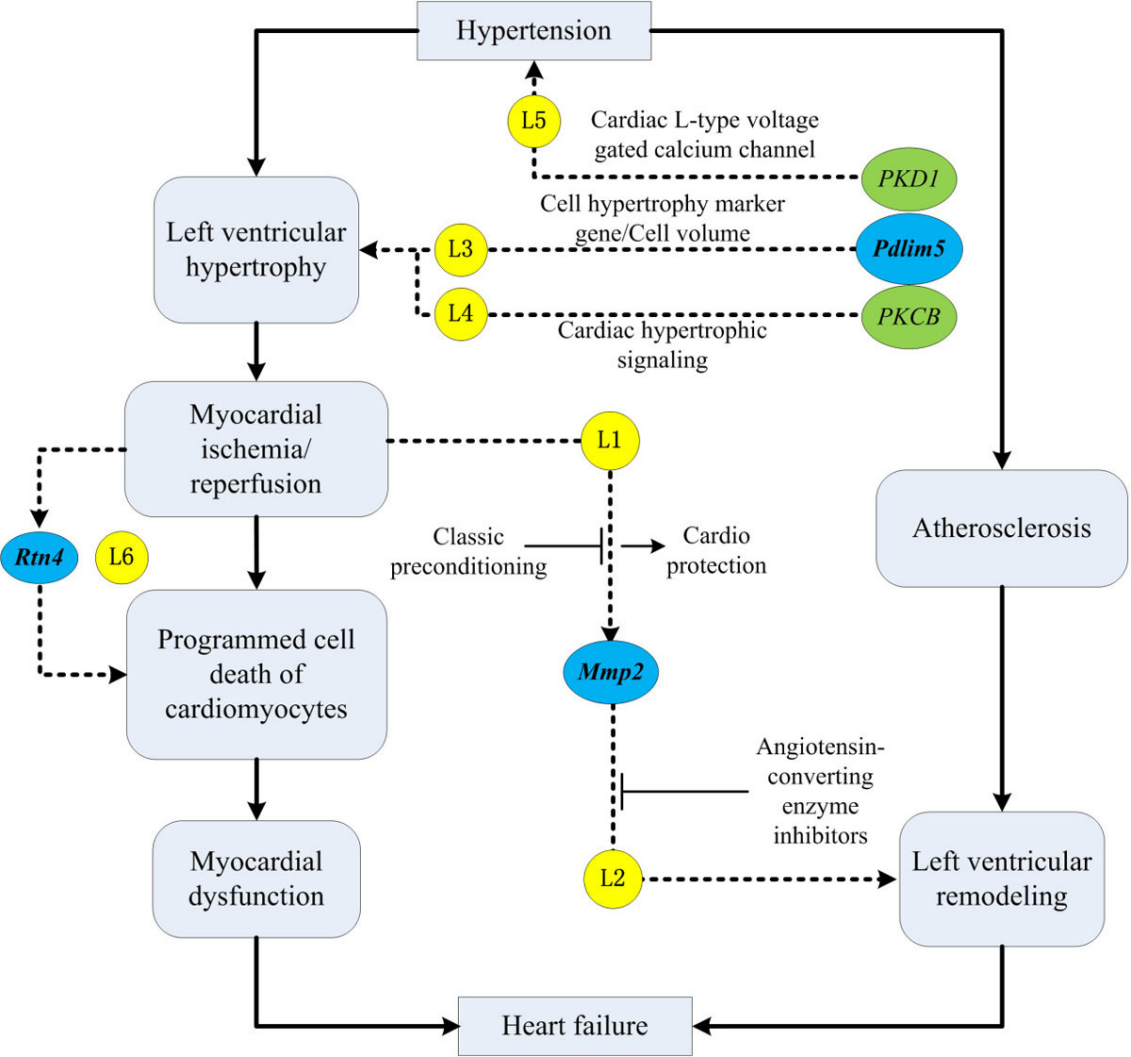
**Literature supports for the results of analyzing GDS3661**. Three genes (*Mmp2, Rtn4 *and *Pdlim5*, marked in blue) identified only by miFDR may perform important roles in the context of hypertensive heart failure. See main text for the detailed explanations of literature evidence labeled as L1-6 (marked in yellow).

We found in literature that several genes identified only by miFDR may shed new light on the molecular mechanism underlying the deterioration of cardio function and remodeling associated with hypertensive heart failure. Figure 4 illustrates the potential roles of three genes (*Mmp2, Rtn4 *and *Pdlim5*) in the context of hypertensive heart failure.

• *Mmp2 *(up-regulated in HF-rats by 1.88 folds) may play a critical role in preventing hypertensive heart failure. On the one hand, it was reported that classic preconditioning can inhibit ischemia/reperfusion induced release and therefore offer cardio protection [23] (Figure 4, **L1**). On the other hand, the suppression of *Mmp2 *activity by angiotensin-converting enzyme inhibitors can prevent left ventricular remodeling in a rat model of heart failure [24] (Figure 4, **L2**). Thus, we hypothesize that inhibiting *Mmp2 *may help prevent heart failure from hypertension.

• *Rtn4 *(up-regulated in HF-rats by 1.59 folds) may have great effects on hypertensive heart failure. Programmed cell death of cardiomyocytes following myocardial ischemia imposes a biomechanical stress on the remaining myocardium, leading to myocardial dysfunction that may cause heart failure or sudden death. It was shown that knocking down *Rtn4 *inhibits the loss of cardiomyocytes following ischemic/hypoxic injury [25]. It was also reported that *Rtn*4 expression was significantly increased in cardiac tissue from patients with dilated cardiomyopathy and from patients who have experienced an ischemic event [26,27] (Figure 4, **L6**). These evidences suggest that myocardial ischemia may trigger *Rtn4*-mediated large scale programmed cell death of cardiomyocytes, which eventually leads to heart failure.

• *Pdlim5 *(up-regulated in HF-rats by 2.96 folds) is a heart and skeletal muscle-specific protein that may perform an essential role in heart development [28]. *Pdlim5 *are related to hypertensive HF in three ways. Firstly, it was reported that *Pdlim5 *promoted the expression of hypertrophy markers and increased cell volume when overexpressed in rat neonatal cardiomyocytes [29] (Figure 4, **L3**). Secondly, *Pdlim5 *protein was found to preferentially interact with protein kinase C *beta *(*PKCB*) which is markedly activated in the cardiac hypertrophic signaling [30] (Figure 4, **L4**). Finally, it was suggested that the protein of *Pdlim5 *scaffolded protein kinase *D*1 (*PKD1*), a key enzyme in the response to stress signals in cardiomyocytes, to regulate the cardiac L-type voltage-gated calcium channels [31] (Figure 4, **L5**). There are several drugs for treating hypertension, myocardial ischemia and cardiac arrhythmias by targeting at this channel.

Besides the three genes (*Mmp2, Rtn4 *and *Pdlim5*) mentioned above, we also found several other interesting genes in literature, such as *Ptgs*1 (up-regulated in HF-rats with 2.06 folds) and *Glrx*2 (up-regulated in HF-rats with 1.35 folds). The human homology of *Ptgs*1 regulates the physiological process involving the growth of new blood vessels from pre-existing vessels in endothelial cells. *Ptgs*1 can mediate endothelial dysfunction under oxidative stress in chronic heart failure [32]. Therefore, *Ptgs*1 may have a strong effect on the onset of hypertensive heart failure.

Mitochondrial *Glrx*2 plays a crucial role in cardio-protection [33]. It was shown that Doxorubicin-induced cardiac injury is reduced in transgenic mice expressing the human *Glrx*2 when compared to non-transgenic mice [34]. Overexpression of human *Glrx*2 in transgenic mice reduces myocardial cell death by preventing both apoptosis and necrosis [33]. We think that the up-regulation of *Glrx*2 is most likely due to the auto-adjustment of the heart system to compensate for heart failure. However, the endogenous mechanisms in those heart failure rats were not able to raise *Glrx*2 up to a level high enough to prevent the onset from happening.

#### GDS3689

At FDR < 0.05, the BH and Storey approaches using ranksum *p*-values failed to identify any significant probe set. If *t*-test *p*-values were used, the BH and Storey approaches identified 18 and 249 significant probe sets, respectively. At the same FDR level, miFDR identified 640 probe sets while SAM only identified 388. We submitted the probe set lists identified by miFDR and SAM for GO enrichment analysis. The result showed that miFDR outperformed SAM in identifying genes in those functional categories closely related to the response to diesel exhaust exposure and hypertension, such as GO-0006952: defense response (miFDR identified 17 genes *vs*. SAM identified 8 genes), GO-0006954: inflammatory response (miFDR 12 genes *vs*. SAM 6 genes), GO-0009967: positive regulation of signal transduction (miFDR 11 genes *vs*. SAM 4 genes), GO-0009968: negative regulation of signal transduction (miFDR 8 genes *vs*. SAM 7 genes), GO-0030198: extracellular matrix organization (miFDR 3 genes *vs*. SAM 0 genes) and GO-0033554: cellular response to stress (miFDR 21 genes *vs*. SAM 10 genes).

Literature evidences also suggested that several genes identified only by miFDR can elucidate new molecular connections between diesel exhaust exposure and hypertension, in particular through atherosclerosis. Atherosclerosis is one of the most serious hypertension-related health problems. The arteries of hypertensive animals have greater mass of vascular smooth muscle than normotensive ones, and the alteration in the differentiated state (e.g. increased proliferation, enhanced migration and down-regulation of vascular smooth muscle differentiation marker genes) of vascular smooth muscle cells is known to perform a key function in the development of atherosclerosis. In addition, diesel exhaust particles greatly promote atherosclerosis [35-37]. One study showed that the synergy between diesel exhaust particles and oxidized phospholipids affect the expression profiles of several gene modules corresponding to the pathways relevant to vascular inflammatory processes such as atherosclerosis [38].

**Figure 5:**
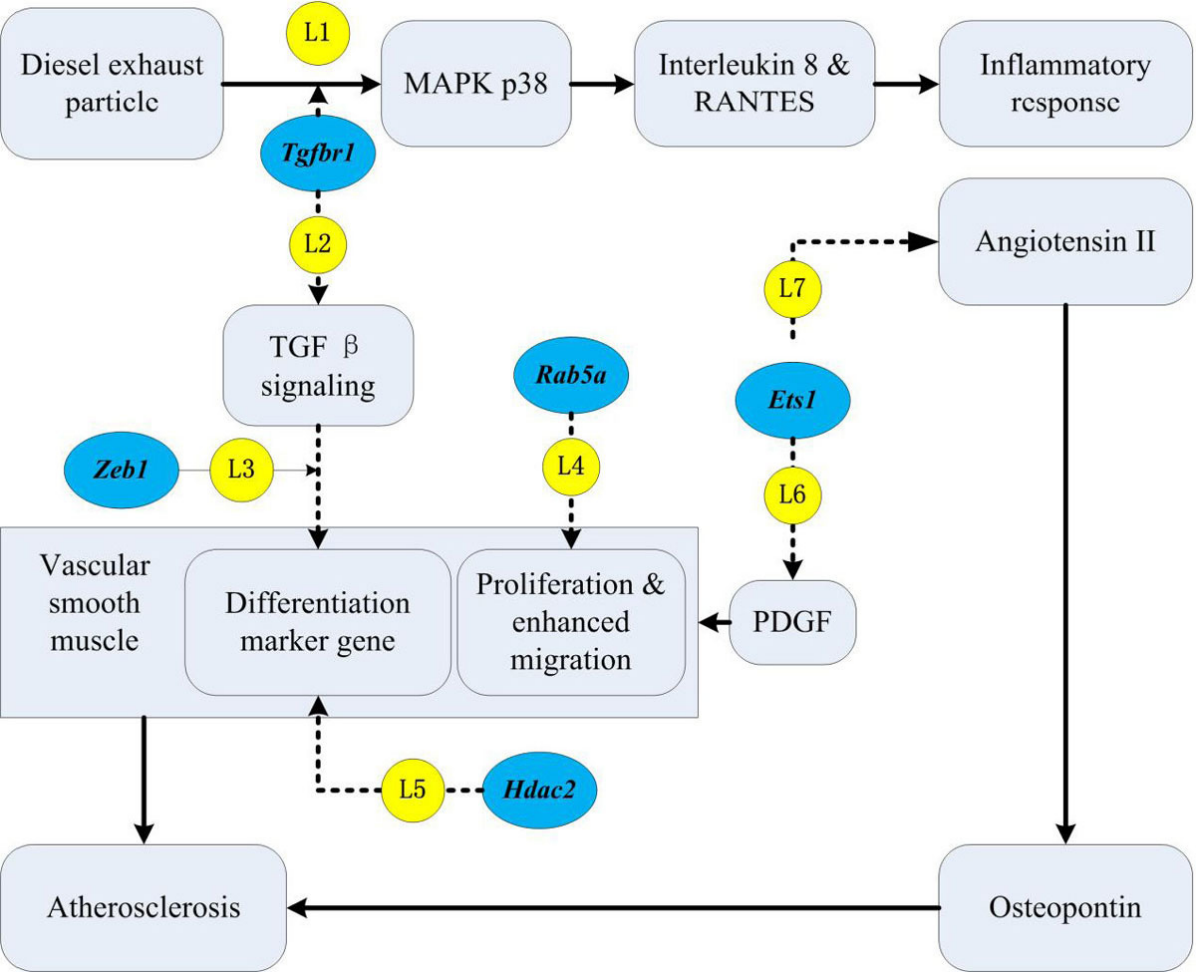
**Literature supports for the results of analyzing GDS3689**. The miFDR approach identified five genes (*Tgfbr1, Zeb1, Hdac2, Rab5a*, and *Ets1*, marked in blue) that may shed new light on the molecular connections between diesel exhaust exposure and hypertension, in particular through atherosclerosis. See main text for detailed explanations of literature evidence labeled as L1-7 (marked in yellow).

Here we focused on five genes (*Tgfbr1, Zeb1, Hdac2, Rab5a*, and *Ets1*), which were all identified by miFDR alone, and discussed their potential roles in the context of diesel particle exposure and atherosclerosis (see Figure 5).

• ***Tgfbr1 ***(up-regulated in DE-rats by 2.05 folds) acts as the upstream of p38 in MAPK signaling pathway (http://www.genome.jp/keggbin/show_pathway?hsa04010). It was shown that diesel exhaust particles activate *p*38 to produce interleukin 8 and RANTES by human bronchial epithelial cells [39]. Thus, we suggest that diesel exhaust particles trigger *p*38 by activating *Tgfbr1 *(Figure 5, **L1**). *Tgfbr1 *also forms a heteromeric receptor complex with *TGF*-*beta *type II receptor that mediates *TGF*-beta signaling (Entrez Gene summary as of May 30th, 2013: http://www.ncbi.nlm.nih.gov/gene/29591) (Figure 5, **L2**).

• ***Zeb*1 **(up-regulated in DE-rats by 1.43 folds) mediates *TGF*-beta signaling in vascular disease and vascular smooth muscle cell differentiation during development [40] (Figure 5, **L3**), which eventually leads to atherosclerosis.

• ***Hdac*2 **(up-regulated in DE-rats by 1.43 folds) was reported to mediate the suppression of vascular smooth muscle cell differentiation marker genes by *POVPC *[1-palmitoyl-2-(5-oxovaleroyl)-sn-glycero-3-phosphocholine] [41]. *POVPC *is concentrated within atherosclerotic lesions and contributes to the pathogenesis of atherosclerosis by inducing profound suppression of vascular smooth muscle cell differentiation marker genes via a transcription factor KLF4 [42] (Figure 5, **L5**).

• ***Rab5a ***(up-regulated in DE-rats by 1.83 folds) was shown to have a strong effect on vascular smooth muscle cell proliferation and migration, which can cause intimal hyperplasia and restenosis. And RNAi-mediated *Rab5a *suppression can inhibit proliferation and migration of vascular smooth muscle cells [43] (Figure 5, **L4**).

• ***Ets*1 **(up-regulated in DE-rats with 1.69 folds) may be related to atherosclerosis in two ways. On the one hand, *Ets*1 was reported to activate platelet-derived growth factor (*PDGF*) *A*-chain [44] and *PDGF D*-chain [45] (Figure 5, **L6**). *PDGF *has been implicated in the pathogenesis of vascular occlusive disorders such as atherosclerosis and restenosis in part due to its regulation of vascular smooth muscle cell phenotype. On the other hand, *Ets*1 is also involved in the signaling mechanisms whereby angiotensin II, a potent up-regulator of osteopontin, increases osteopontin expression in vascular smooth muscle cells [46] (Figure 5, **L5**). Several recent studies have revealed that osteopontin performed multiple roles in the progression of atherosclerotic plaques [47-51].

## Conclusions

We presented a new powerful FDR control method - miFDR, which minimizes the estimated FDR when calling a fixed number of significant features. We showed theoretically that the search strategy of miFDR maximizes findings given any certain FDR cut-off. We validated this idea by showing that miFDR outperformed the other three widely accepted FDR control methods (SAM, BH and Storey) in simulation tests and DNA microarray analysis. Literature evidences support that several genes identified only by miFDR are indeed relevant to the underlying biology of interest. Controlling FDR is critical in analyzing genome-wide datasets. Therefore, miFDR is an important innovation that will benefit projects utilizing high-throughput technologies and make a broad impact in the future.

## Competing interests

The authors declare that they have no competing interests.

## Authors' contributions

Both authors contributed equally to this work.

## Supplementary Material

Additional file 1 The supplementary materials contain Appendices A, B, and C. Appendices A and B show the simulation test results using different sample sizes: 6 *vs*. 6 and 10 *vs*. 10, respectively. Appendix C proves a theorem that allows us to automatically decide the upper-bound of *M_ρ _*in Algorithm 2 for a given FDR cut-off. Click here for file

## Declarations

The publication costs for this article were funded by the corresponding author.

This article has been published as part of *BMC Systems Biology *Volume 7 Supplement 4, 2013: Selected articles from the IEEE International Conference on Bioinformatics and Biomedicine 2012: Systems Biology. The full contents of the supplement are available online at http://www.biomedcentral.com/bmcsystbiol/supplements/7/S4.
