# Supplementary material for: A novel approach to minimize false discovery rate in genome-wide data analysis
Source: BMC Syst Biol. 2013 Oct 23;7(Suppl 4):S1. doi: 10.1186/1752-0509-7-S4-S1 (PMC3856609; doi:10.1186/1752-0509-7-S4-S1)

A Novel Approach to Minimize False Discovery Rate in Genome-Wide Data Analysis – Supplementary materials

Yuanzhe Bei[1]*, Pengyu Hong [1]*§

[1] Department of Computer Science, Brandeis University, 415 South St., MA 02453, USA

*These authors contributed equally to this work

§Corresponding author

Email addresses:

YB: beiyz@brandeis.edu

PH: hongpeng@brandeis.edu

Appendix A

We carried out a simulation test similar to the one presented in the Results section of the main paper except that the simulated dataset contained 12 samples (6 in the 1st group and the other 6 in the 2nd group). We ran the simulation test 1000 times. Each time we obtained a curve for each of the four approaches (miFDR, SAM, BH, and Storey) showing the estimated FDRs *vs.* the numbers of features called significant. We then calculated the mean curve of each approach with respect to the number of features called significant.

Figure S1 shows that miFDR consistently called more significant features than SAM did at the same estimated FDR levels. Under the FDR cut-off = 0.05, miFDR on average identified 7.19% more features than SAM did (7.223 *vs* 6.704 with a paired *t*-test p = 5.51e-67). The estimated FDRs and the true FDRs of those approaches are compared in **Figure S2**, which shows that miFDR is the best performer. True FDR was calculated as the ratio between the number of null features falsely called significant and the total number of features called significant. **Figure S3** shows that the true FDR of miFDR is well bounded by its estimated FDR.

The BH and Storey approaches performed much worse than both miFDR and SAM (**Figure S1 & S2**). They consistently called fewer numbers of features than miFDR and SAM did at the same estimated FDR levels. In addition, their true FDRs are significantly higher than their estimated FDRs, which we think mainly due to the following reason. Fifty percent of features follow Gaussian distributions, and the other fifty percent follow uniform distributions. However, the BH and Storey approach using *t*-test to calculate *p*-values, which assumes Gaussian distributions. We also tried using

the ranksum *p*-values in the BH and Storey approaches. Nevertheless, the performances were even worse (see **Figure S4**).

Appendix B

We carried out a simulation test similar to the one presented in the Results section of the main paper except that the simulated dataset contained 20 samples, 10 of which are in the 1st group and the other 10 are in the 2nd group. The experiment contains 1000 simulation runs in total. Each time we obtained a curve for each of the four approaches (miFDR, SAM, BH, and Storey) showing the estimated FDRs *vs.* the numbers of features called significant. We then calculated the mean curve of each approach with respect to the number of features called significant.

We observed that miFDR consistently called more significant features than SAM did at the same estimated FDR levels (see **Figure S5**). For example, at FDR cut-off = 0.05, miFDR on average identifies 22.18% more features than SAM did (62.19 *vs* 48.40 with a paired *t*-test p = 3.78e-181). Both the estimated and true FDRs of those approaches are shown in **Figure S6**. Again miFDR performed the best among all four approaches. **Figure S7** shows that the true FDR of miFDR is well bounded by its estimated FDR.

The BH + *t*-test and Storey + *t*-test approaches performed much worse than both miFDR and SAM (**Figure S5 & S6**). They consistently called fewer numbers of features than miFDR and SAM did at the same estimated FDR levels. In addition, their true FDRs are significantly higher than their estimated FDRs. We also tried using the

ranksum *p*-values in the BH and Storey approaches. Nevertheless, the performances were even worse (see **Figure S8**).

Appendix C

In this appendix, we prove a theory that allows us to automatically decide the upper-bound of M_ρ for a given FDR cut-off.

Let $FDR^+(N^+)$ denote the one-sided FDR for calling N^+ positive significant features

$$FDR^+(N^+) = \frac{FP^+(N^+)}{N^+} \cdot P(H = 0)$$

(s1)

where $FP^+(N^+)$ is the number of false significant features when calling N^+ positive significant features.

Let $FDR^-(N^-)$ denote the one-sided FDR for calling N^- negative significant features

$$FDR^-(N^-) = \frac{FP^-(N^-)}{N^-} \cdot P(H = 0)$$

(s2)

where $FP^-(N^-)$ is the number of false significant features when calling N^- negative significant features.

Let $FDR(N^+, N^-)$ denote the overall FDR for N^+ positive significant features and N^- negative significant features

$$FDR(N^+, N^-) = \frac{FP^+(N^+) + FP^-(N^-)}{N^+ + N^-} \cdot P(H = 0)$$

(s3)

Theorem S1: Given a FDR cut-off $\Psi < 0.5$ and a particular $N_0 > 0$, if $FDR^+(N_0) \geq 2\Psi$ and $FDR^-(N_0) \geq 2\Psi$, then for $\forall N^+ \geq 0$ & $N^- \geq 0$ that satisfies $N^+ + N^- = 2N_0$, we have $FDR(N^+, N^-) \geq \Psi$.

Proof:

By definition of *FDR*$^{+}$ and *FDR*$^{-}$, we have

$$FDR^{+}(N_0) \geq 2\Psi \quad \Longrightarrow \quad \frac{FP^{+}(N_0)}{N_0} \cdot P(H=0) \geq 2\Psi \tag{s4.1}$$

$$FDR^{-}(N_0) \geq 2\Psi \quad \Longrightarrow \quad \frac{FP^{-}(N_0)}{N_0} \cdot P(H=0) \geq 2\Psi \tag{s4.2}$$

$N^{+} + N^{-} = 2N_0$ & $N^{+} \geq 0$ & $N^{-} \geq 0 \;\; \Longrightarrow \;\; N^{+} \geq N_0$ or $N^{-} \geq N_0$.

We can prove that, for both cases, the theorem holds.

(a) $N^{+} \geq N_0$.

$$N^{+} \geq N_0 \quad \Longrightarrow \quad FP^{+}(N^{+}) \geq FP^{+}(N_0)$$

$$\Longrightarrow \quad \frac{FP^{+}(N^{+})}{N_0} \cdot P(H=0) \geq \frac{FP^{+}(N_0)}{N_0} \cdot P(H=0) = FDR^{+}(N_0) = 2\Psi$$

$$\Longrightarrow FP^{+}(N^{+}) \geq \frac{2\Psi}{P(H=0)} \cdot N_0 = \frac{2\Psi}{P(H=0)} \cdot \frac{N^{+}+N^{-}}{2} = \frac{\Psi \cdot (N^{+}+N^{-})}{P(H=0)}$$

(s5)

Since $FP^{-}(N^{-}) \geq 0$, plugging eq. (s3) into eq. (s5), we have

$$FP^{+}(N^{+}) + FP^{-}(N^{-}) \geq FP^{+}(N^{+}) \geq \frac{\Psi \cdot (N^{+}+N^{-})}{P(H=0)}$$

$$\Longrightarrow \frac{FP^{+}(N^{+}) + FP^{-}(N^{-})}{(N^{+}+N^{-})} \cdot P(H=0) \geq \Psi$$

$$\Longrightarrow FDR(N^{+}, N^{-}) \geq \Psi$$

(s6)

(b) $N^{-} \geq N_0$

$$\frac{FP^{-}(N^{-})}{N_0} \cdot P(H=0) \geq \frac{FP^{-}(N_0)}{N_0} \cdot P(H=0) = FDR^{-}(N_0) = 2\Psi$$

$$\Longrightarrow FP^{-}(N^{-}) \geq \frac{2\Psi}{P(H=0)} \cdot N_0 = \frac{2\Psi}{P(H=0)} \cdot \frac{N^{+}+N^{-}}{2} = \frac{\Psi \cdot (N^{+}+N^{-})}{P(H=0)}$$

$$\xrightarrow{FP^+(N^+)\geq 0} FP^-(N^-) + FP^+(N^+) \geq FP^-(N^-) \geq \frac{\Psi \cdot (N^+ + N^-)}{P(H=0)}$$

$$\Rightarrow \frac{FP^-(N^-) + FP^+(N^+)}{(N^+ + N^-)} \cdot P(H=0) \geq \Psi$$

$$\Rightarrow FDR(N^+, N^-) \geq \Psi$$

(s7)

■

According to Theorem S1, given a FDR cut-off Ψ, we can decide M_ρ efficiently as follows. First find the minimum $N_0^* > 0$ that satisfy for $\forall a \geq N_0^*$ we have $FDR^+(a) \geq 2\Psi$ and $FDR^-(a) \geq 2\Psi$. Then set $M_\rho = 2N_0^*$. The best N^+ and N^- can be obtained by miFDR must be bounded by M_ρ. Below we show that N_0^* can also be found efficiently.

For any $N_0 > 0$ that satisfy for $FDR^+(N_0) \geq 2\Psi$, we can efficiently know whether $\exists a \geq N_0$, *s.t.* $FDR^+(a) < \Psi$ as follows. For $\forall a \geq N_0$, its $FDR^+(a)$ can be written as

$$FDR^+(a) = \frac{FP^+(a)}{a} \cdot P(H=0)$$

$$= \frac{FP^+(a)}{FP^+(N_0)} \cdot \frac{N_0}{a} \cdot \frac{FP^+(N_0)}{N_0} \cdot P(H=0)$$

$$= FDR^+(N_0) \cdot \frac{FP^+(a)}{FP^+(N_0)} \cdot \frac{N_0}{a}$$

$$\xrightarrow{FP^+(a)\geq FP^+(N_0)} FDR^+(a) \geq FDR^+(N_0)\frac{N_0}{a}$$

(s8)

Let $N_1 = g(N_0) = \max(\left\lceil \frac{FDR^+(N_0)}{2\Psi} N_0 \right\rceil, N_0 + 1)$. For $\forall a$ *s.t.* $N_0 < a < N_1$, according to eq. (s8), we have $FDR^+(a) \geq 2\Psi$ because

$$FDR^{+}(a) \geq FDR^{+}(N_0)\frac{N_0}{a} \geq FDR^{+}(N_0)\frac{N_0}{N_1 - 1}$$

(s9)

If $N_1 = \left\lceil \frac{FDR^{+}(N_0)}{2\Psi} N_0 \right\rceil$, we have

$$FDR^{+}(a) \geq FDR^{+}(N_0)\frac{N_0}{\frac{FDR^{+}(N_0)}{2\Psi} N_0} = 2\Psi$$

Otherwise if $N_1 = N_0 + 1$, we have

$$FDR^{+}(a) \geq FDR^{+}(N_0)\frac{N_0}{N_0 + 1 - 1} = FDR^{+}(N_0) \geq 2\Psi$$

Therefore, once find N_0, we can generate the following series of numbers recursively: $N_1 = g(N_0)$, $N_2 = g(N_1)$, $N_3 = g(N_2)$, …, until it hits the total number of features M. We only need to find the largest N_i that satisfy $FDR^{+}(N_i) < 2\Psi$ and $FDR^{+}(N_{i+1}) \geq 2\Psi$, and then set $N_0^{*} = N_{i+1}$. In practice, we often require $\Psi \leq 0.1$. In the null hypothesis territory, FDR increases very quickly towards 1 so that N_i will increase exponentially with respect to i towards M. Therefore, the above procedure for finding N_0^{*} is very efficient. The same idea can be applied to ensure that N_0^{*} works for FDR^{-}.

Figures

Figure S1 - Compare the estimated FDRs(6 *vs.* 6)

Compare the average estimated FDR of BH, Storey, SAM and miFDR on 1000 simulation runs (6 *vs.* 6). A blow-out of the curve segment in the dash rectangle is shown at the bottom-right corner for clearer illustration. The performance of miFDR is the best.

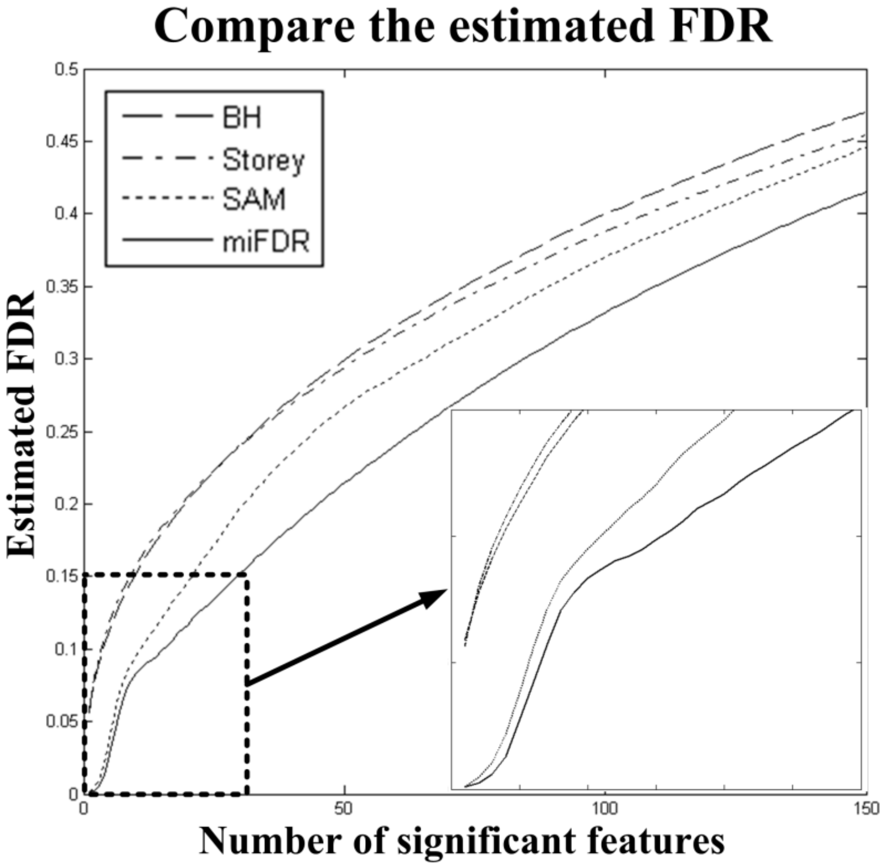

Figure S2 - Compare the true FDRs (6 *vs.* 6)

Compare the averaged true FDR of BH, Storey, SAM and miFDR ove 1000 simulation runs (6 *vs.* 6). A blow-out of the curve segment in the dash rectangle is shown at the bottom-right corner for clearer illustration. The performance of miFDR is the best.

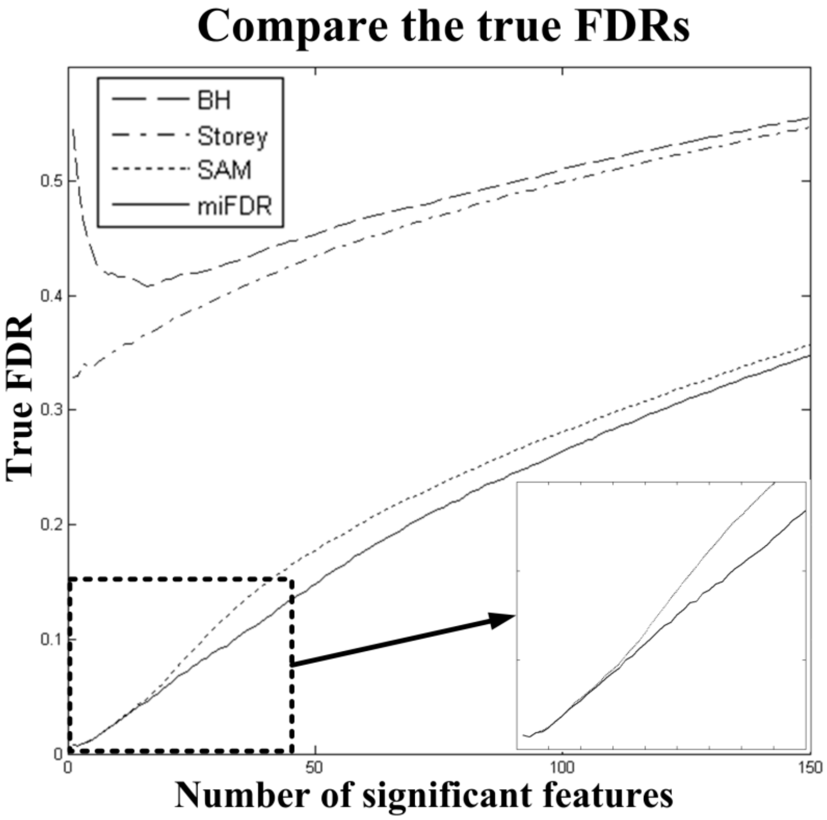

Figure S3 - Compare the true and estimated FDRs of miFDR (6 *vs.* 6)

Compare the averaged true and estimated FDRs of miFDR over 1000 simulation runs. A blow-out of the curve segment in the dash rectangle is shown at the bottom-right corner for clearer illustration. The true FDR of miFDR is bounded by its estimated FDR.

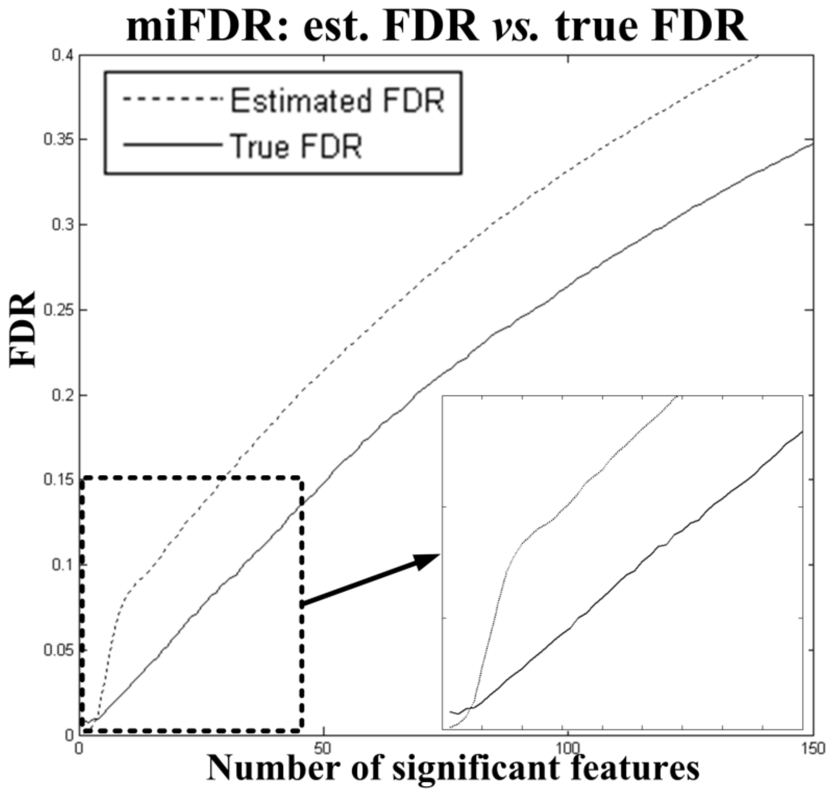

Figure S4 - Compare the performances of BH and Storey using ranksum test and *t*-test (6 *vs.* 6)

Compare the average performances of BH + *t*-test, Storey + *t*-test, BH + ranksum, Storey + ranksum on the simulated datasets (6 *vs.* 6). The performances of BH + *t*-test and Storey + *t*-test are much better than those of BH + ranksum and Storey + ranksum.

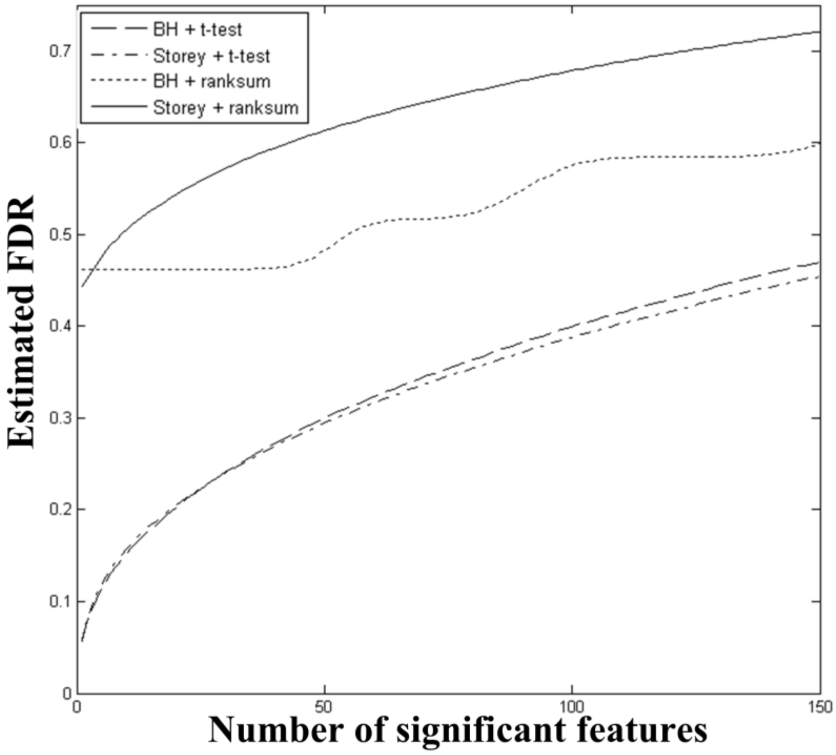

Figure S5 - Compare the estimated FDRs (10 *vs.* 10)

Compare the estimated FDRs of BH, Storey, SAM and miFDR that are averaged over 1000 simulation runs. A blow-out of the curve segment in the dash rectangle is shown at the upper-left corner for clearer illustration. The performance of miFDR is the best.

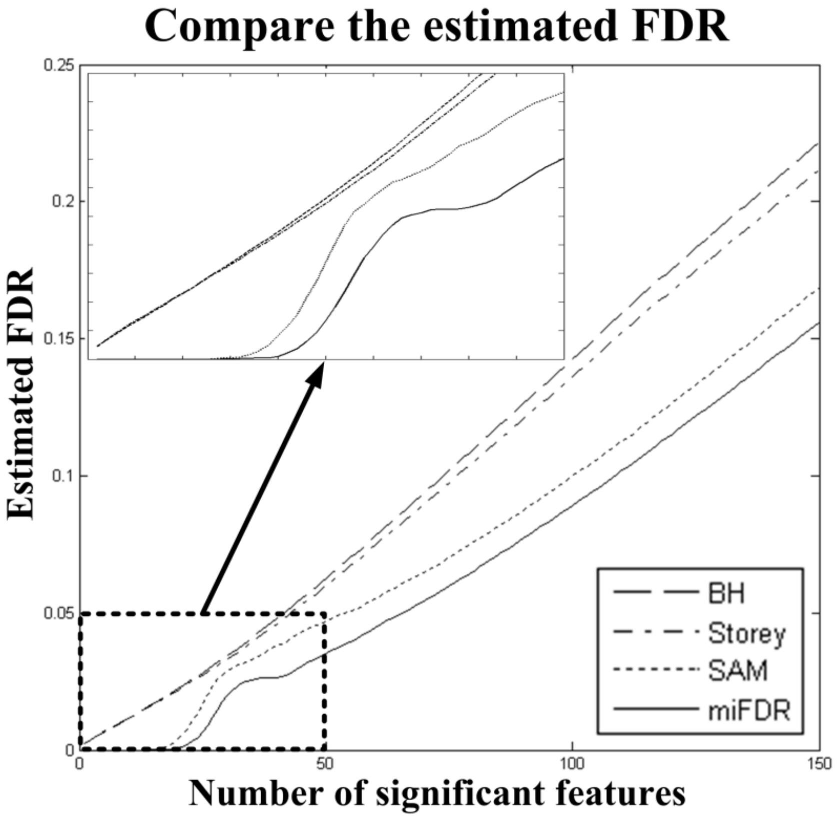

Figure S6 - Compare the true FDRs (10 *vs.* 10)

Compare the averaged true FDRs of BH, Storey, SAM and miFDR over 1000 simulation runs. A blow-out of the curve segment in the dash rectangle is shown at the upper-left corner for clearer illustration. The performance of miFDR is the best.

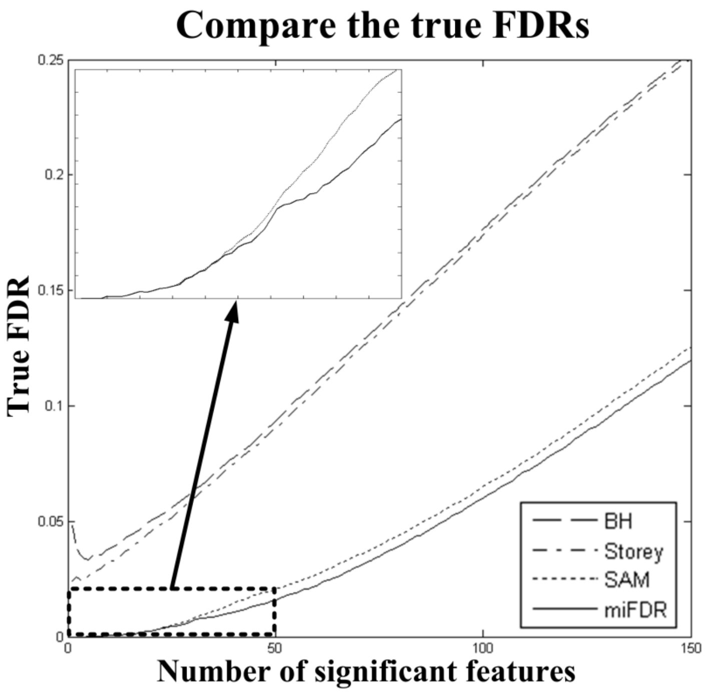

Figure S7 - Compare the true and estimated FDRs of miFDR (10 *vs.* 10)

Compare the averaged true and estimated FDR of miFDR over 1000 simulation runs. A blow-out of the curve segment in the dash rectangle is shown at the upper-left corner for clearer illustration. The true FDR of miFDR is bounded by its estimated FDR.

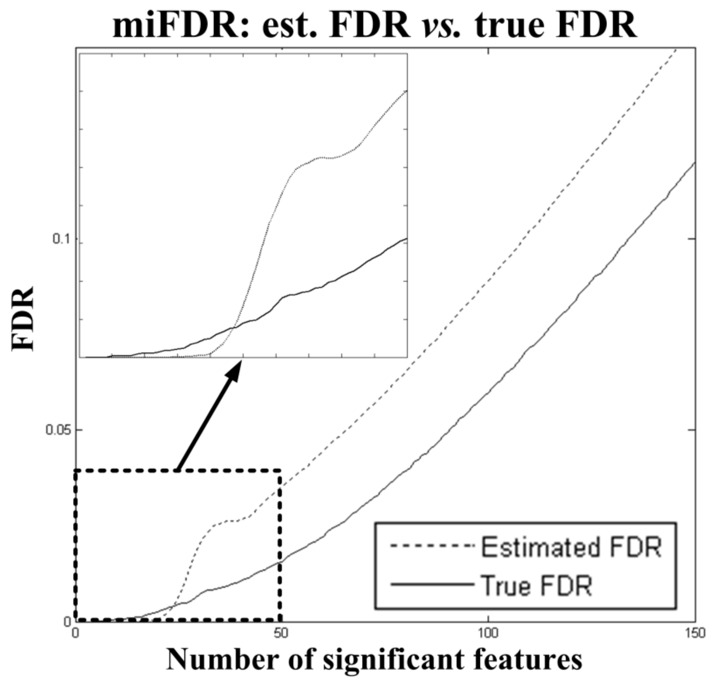

Figure S8 - Compare the performances of BH and Storey using ranksum test and *t*-test (10 *vs.* 10)

Compare the averaged performances of BH + *t*-test, Storey + *t*-test, BH + ranksum, Storey + ranksum using 1000 simulation runs. The performances of BH + *t*-test and Storey + *t*-test are much better than those of BH + ranksum and Storey + ranksum.

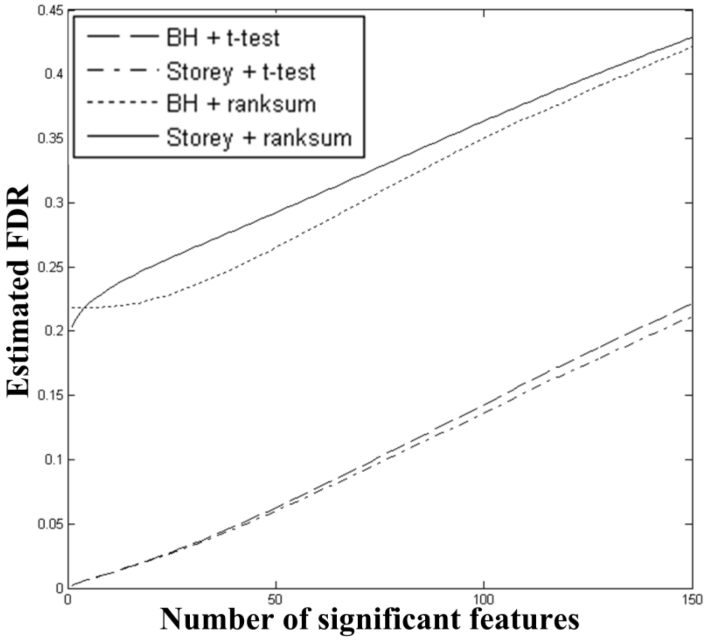

Supplement: Additional file 1 — The supplementary materials contain Appendices A, B, and C. Appendices A and B show the simulation test results using different sample sizes: 6 vs. 6 and 10 vs. 10, respectively. Appendix C proves a theorem that allows us to automatically decide the upper-bound of Mρ in Algorithm 2 for a given FDR cut-off. [file 1752-0509-7-S4-S1-S1.pdf]
